# Edible Mushrooms as a Natural Source of Food Ingredient/Additive Replacer

**DOI:** 10.3390/foods10112687

**Published:** 2021-11-03

**Authors:** Esmeralda Rangel-Vargas, Jose Antonio Rodriguez, Rubén Domínguez, José Manuel Lorenzo, Maria Elena Sosa, Silvina Cecilia Andrés, Marcelo Rosmini, José Angel Pérez-Alvarez, Alfredo Teixeira, Eva María Santos

**Affiliations:** 1 Área Académica de Química, Universidad Autónoma del Estado de Hidalgo, Ctra. Pachuca-Tulancingo Km 4.5 s/n, Col. Carboneras, Mineral de la Reforma 42183, Hidalgo, Mexico; esmeralda_rangel10403@uaeh.edu.mx (E.R.-V.); josear@uaeh.edu.mx (J.A.R.); 2 Centro Tecnológico de la Carne de Galicia, Rúa Galicia n° 4, Parque Tecnológico de Galicia, San Cibrao das Viñas, 32900 Ourense, Spain; rubendominguez@ceteca.net; 3 Área de Tecnología de los Alimentos, Facultad de Ciencias de Ourense, Universidad de Vigo, 32004 Ourense, Spain; 4 Departamento de Alimentos, Campus Irapuato-Salamanca, Universidad de Guanajuato, Ex-Hacienda El Copal, Carretera Irapuato-Silao km 9, Irapuato 36500, Guanajuato, Mexico; msosa@ugto.mx; 5 Centro de Investigación y Desarrollo en Criotecnología de Alimentos (CIDCA, CONICET-CICPBA-UNLP), Facultad de Ciencias Exactas, UNLP, 47 y 116, La Plata 1900, Argentina; scandres@biol.unlp.edu.ar; 6 Department of Public Health, Faculty of Veterinary Science, National University of Litoral, Esperanza 3080, Argentina; mrosmini@unl.edu.ar; 7 IPOA Research Group, Agro-Food Technology Department, Orihuela Polytechnical High School, Environmental and Agrofood Research Centre for Research and Innovation (CIAGRO), Universidad Miguel Hernández de Elche, 03312 Orihuela, Alicante, Spain; ja.perez@umh.es; 8 Centro de Investigação de Montanha (CIMO), Instituto Politécnico de Bragança, Campus de Santa Apolónia, 5300-253 Bragança, Portugal; teixeira@ipb.pt

**Keywords:** meat, flour, fat substitution, salt replacer, antioxidant, healthier foods, natural additive

## Abstract

Although mushrooms have been exploited since ancient times because of their particular taste and therapeutic properties, the interest in edible species as a source of ingredients and bioactive compounds is recent. Their valuable nutritional contents in protein, dietary fiber and bioactive compounds make them ideal candidates for use in foods in efforts to improve their nutritional profiles. This trend is in line with the consumer’s growing demand for more plant-based foods. The present review paper explores different studies focused on the use of common edible mushrooms as an ingredient and additive replacer by using them in fresh, dried, or even extract forms, as meat, fat, flour, salt, phosphates, and antioxidant replacers. The replacement of meat, fat, flour, and salt by mushrooms from commercial species has been successful despite sensorial and textural parameters can be affected. Moderate concentrations of mushrooms, especially in powder form, should be considered, particularly in non-familiarized consumers. In the case of antioxidant and antimicrobial properties, results are variable, and more studies are necessary to determine the chemical aspects involved.

## 1. Introduction

Food consumption has evolved from the acquisition of essential macro and micronutrients to cover metabolic needs to a conscious activity, which can affect our health in a positive or negative way [1]. In light of this growing tendency, some foods, ingredients, and additives have become health villains when they are over-consumed, such as fat, salt, meat origin products, synthetic substances, including preservatives and antioxidants. Furthermore, sustainable issues especially focused on greenhouse gas emissions from animal protein production, have driven a shift in the consumer’s behavior towards an increasing demand for plant-based products as “better for you” and “better for the planet” alternatives [2]. In this sense, there has been an explosion in the last decades in the quest for new natural sources of ingredients/additives to replace the above-mentioned and provide the consumer with healthier foods [3]. Proteins and dietary fibers from underutilized or byproducts from vegetables, pulses, cereals, tubers, edible seeds, or even unexpectable sources, such as algae or insects, have been studied as a source of ingredient or additive to improve the nutritional and functional properties of food [4,5,6].

Edible mushrooms are one of the recently rediscovered sources of several functional ingredients [7,8]. They are known from ancient times and Romans regarded mushrooms as the “Food of Gods” [9]. Highly exploited by Asian countries because of their nutraceutical and therapeutic properties, China is the world’s largest producer of edible mushrooms and truffles accounting for over 70% of the world’s production [10]. There are at least 14,000 known species of mushrooms of which 2000 are safe and suitable for edible and/or medicinal application, although only 35 are currently commercially explored [11]. The widely common mushroom species produced in suitable ecological conditions are *Agaricus* spp. (champignon), *Lentinula edodes* (shiitake), *Pleurotus* spp. (oyster), *Volvariella volvacea* (straw), *Auricularia auricula* (wood ear), *Ganoderma* (Reishi), *Grifola frondosa* (maitake), *Flammulina velutipes* (winter) and *Tremella* (white jelly) [12].

Mushrooms are categorized as a healthy food because they are low in calories and fat, but rich in proteins, dietary fiber (chitin, hemicellulose, mannans, and β-glucans), and minerals [13,14]. All essential amino acids are present in mushrooms and polyunsaturated fatty acids are in higher proportion compared to saturated fatty acids [15]. Their chemical profiles include several bioactive compounds, such as polysaccharides, biologically active proteins (enzymes, lectins), ergothioneine (amino acid), terpenoids, sterols, antioxidants, and vitamins (thiamine, riboflavin, ascorbic acid, niacin, and tocopherols), which contribute to improved immune function, as well as presenting antitumor, antimicrobial, anti-inflammatory, hypoglycemic, hypocholesterolemic and antioxidant effects [12,16].

Their nutritional value, antioxidant activity, and health-beneficial properties, as well as the flavor and texture properties, have attracted the industry’s attention to use mushrooms as possible substitutes for several ingredients and additives in processed foods, mainly bakery and meat products [17]. In this work, studies from the last decade about the use of edible mushrooms as a food ingredient or additive replacer in foods are reviewed.

## 2. Edible Mushrooms as Ingredient/Additive Replacers

### 2.1. Meat Replacer

The interesting content of protein (19–40% on dry basis), dietary fiber in mushrooms [14,15,18,19], as well as the umami flavor and fibrous texture, has encouraged their use to replace animal protein in meat products [8]. Edible mushrooms are significant sources of protein, compared to most vegetables, accounting the eight essential amino acids the 25–45% of the total amino acids [14,19]. However, protein content is variable depending on mushroom species and growing conditions [15]. Despite the low amount of fat in mushrooms (2–6% on dry basis), the unsaturated oleic and linoleic acids are the predominant [15]. The mushroom dietary fiber is the edible part of the mushroom that is resistant to digestion and absorption in the human small intestine. Dietary fiber in mushrooms comprises chitin, hemicellulose, mannans, and β-glucans [14,19]. According to Hussein et al. [6] dietary fiber ranges 27–44% on dry basis, being soluble fiber around 10% of the total fiber. The substitution of meat by mushrooms fits with the growing need for a shift from a meat-centered diet to a plant-based diet, which encourages consumption of meat less frequently and/or in smaller portions, proposed by Spencer and Guinard [20] as the Flexitarian Flip™.

For this purpose, the use of washed, scalded/cooked and ground mushrooms as blends has been the simple way to reduce the meat content as blends. Different studies have partially replaced meat in patty formulations (chicken, beef, or pork) by the use of *Pleurotus sajor caju* [21,22], *Tremella fuciformis* [23], and *Agaricus bisporus* [24] (Table 1). *Agaricus bisporus* was the potential meat replacer in meat-based dishes (*carne asada* and *taco* blends) with meat substitutions between 50–80% [25,26]. Wong et al. [27] reduced up to 45% of meat in taco filling with blanched and ground *A. bisporus*. This mushroom species was also considered to replace 15% of sutchi catfish in fish patties with good antioxidant and antimicrobial properties since patties with mushrooms remained acceptable even after 16 days of storage comparing to control samples, which were spoiled at that time [28]. *Pleurotus eryngii* is another species used in the replacement of 20 to 50% of cuttlefish meat to elaborate surimi gel [29]. The incorporation of mushrooms as blends usually gives softer products, since an increase in moisture is expected considering the high water content of the mushrooms [29,30]. Higher cooking yields (less water and fat cooking losses of the product during cooking) are also associated with this substitution because of the higher binding capacity of mushrooms [23]. Despite the textural modifications, the previously mentioned meat products were well accepted with good liking scores, especially where moderate percentages of meat were substituted with the extra bonus of adding dietary fiber, mainly insoluble dietary fiber.

One of the limitations for the use of mushrooms as blends is that they are highly perishable because of the high water content and strong postharvest respiration [31]. Thus, a drying procedure can be an option to extend the shelf-life of mushrooms when they are used as alternative ingredients. Drying is one of the traditional methods used in edible fungi processing, and both natural drying and technology-assisted drying (e.g., solar drying, spray drying, hot air drying, vacuum drying, microwave drying, and freeze-drying) are commonly used [32]. Nevertheless, the drying methods applied to extend the shelf-life of the mushrooms can alter not only their nutritional value but also the taste and bioactive properties [33,34]. The concentration of heavy metals should also be studied in the case of dried wild mushrooms, where growing substrates are not controlled, since mushroom fruiting bodies are prone to bioaccumulation of heavy metals [35].

When meat substitution have been carried out by the incorporation of dried mushroom, lower percentages of meat substitutions have usually been tested. For example, the replacement of 2%, 4%, and 6% of meat with *Pleurotus sajor-caju* powder was tested in chicken Frankfurters with an increase in dietary fiber to 6.20% [36]. Dried *Lentinula edodes* has been another mushroom species used as a potential meat substitute in pork meat sausages with acceptable sensory properties at 25% of replacement [37]. Both works reported an increase in dietary fiber content with slight texture modifications, mainly a hardness decrease. The diminution of hardness is attributed to the reduction of protein content by meat reduction, affecting meat emulsion [37].

The replacement of 2–6% of meat by *Flammulina velutipes* steam in goat meat nuggets has led to an increase in water holding capacity with similar protein content. The dried powder absorbed water to hydrate itself and helped to reduce the cooking losses and producing softer nuggets [38]. On the contrary, Wan-Mohtar et al. [39] observed an increase in texture after replacing meat by dried ground *Pleurotus sapidus* in chicken nuggets. For these authors, a 10% replacement of meat by mushroom flour was recommended to avoid excessive hardness, dark color, and unpleasant taste derived from higher mushroom concentrations [39].

### 2.2. Fat Replacer

Animal fat can reach levels over 30% in some meat products, which is a limiting factor for those consumers who are more aware of the relationship between diet and health. According to the World Health Organization (WHO) [40], total fat intake should be less than 30% of total energy intake in order to achieve an adequate nutritional balance and prevent malnutrition and some metabolic diseases. Presently, total or partial replacement of fat in meat products has attracted attention from consumers and the industry [41,42]. However, the reduction of fat in meat products can lead to a possible rejection because of the negative impact on sensory properties. Several successful attempts have been made to substitute animal fat with edible mushrooms [17,43,44,45,46], reducing the caloric content in meat products (Table 1). Wang et al. [43] replaced the total pork back fat in the elaboration of pork sausage with *Pleurotus eryngii* prepared in different presentations (raw, boiled, deep-fried, and fried) showing the best sensory results with the use of deep-fried and fried mushrooms. Additionally, apart from the expected reduction of fat, an increase in protein and fiber content was noticed, as well as an increase in water holding capacity attributed to the higher moisture [43]. The substitution of 30% and 50% of fat with *Agaricus bisporus* and *Pleourotus ostreatus* powder (2.5% and 5%) was tested in frankfurters with similar results regarding increased protein and fiber contents [44]. Sausages with mushrooms added were softer, especially in the *Pleourotus* added samples, and the samples were considered sensorially acceptable, although control samples presented higher acceptance. In general, when the reduction of fat is compensated by increasing water and maintaining protein content, the structure of low-fat systems becomes softer [44]. A reduction of 30% of fat by adding 2.5% of mushroom powder was considered the most feasible to familiarize the consumers with the new flavor.

Burgers are other meat products with high contents of fat [47,48]. Patinho et al. [17] replaced up to 75% of fat by cooked *Agaricus bisporus* in beef burgers, obtaining good overall liking scores for the product that was characterized as juicy, tender, and flavor, some with less cooking loss. According to the authors, the high water and moisture retention of mushrooms helps to reduce the cooking losses by water lost [17]. In a previous work of the same authors [49], the reduction of fat was combined with salt reduction up to 75% and similar results were obtained. Ceron-Guevara et al. [45] used *Agaricus bisporus* as well as *Pleurotus ostreatus* (2.5% and 5%) to replace 50% of animal fat in beef burgers. Since the mushrooms were added as a powder, in this case, products with 2.5% of mushroom flour were more sensorially accepted. In fact, *Agaricus bisporus* affects the product’s color, thus providing darker products by decreasing the luminosity values. Regarding texture, the inclusion of mushroom powder compensated for the reduction of fat avoiding the excessive increase in hardness. Cerón-Guevera et al. [46] also tested these mushroom species in liver pate with the same purpose (fat and salt reduction). Pate is a high caloric meat product with fat content from 25–45% [50]. The replacement of fat by the mushroom flours increased hardness, adhesiveness, and gumminess by modifying protein–water and protein–protein gel network, increasing the gel consistency.

### 2.3. Flour Replacer

The drying and grounding preservation techniques make the mushrooms especially suitable to be incorporated in pasta, bakery, or snack products as flour ingredient (Table 1). Several works have replaced wheat flour with different mushroom powders. For example, 1 to 3% of β-glucans from *Lentinus edodes* in bakery foods [51], 5–15% of chestnut mushroom (*Agrocybe aegerita*) in extruded snacks [52], 5–15% of *Pleurotus sajor-caju* in biscuits [53], 10–30% of *Lentinula edodes* in cookies and steam buns [54], or 4–12% of *Pleurotus sajor caju* in biscuits [55]. *Pleurotus ostreatus* has also successfully replaced wheat flour in noodles [56,57]. In general, the introduction of mushroom flour impoverishes pasting properties, providing darker products and adding firmness. The inclusion of mushroom fibers also weakens the gluten network [53,55]. According to Yuan et al. [58], substitution of a maximum of 5% of wheat flour by *Auricularia auricula* powder still offered an acceptable gluten network microstructure without affecting the functionality of the wheat dough. On the other hand, an increase in dietary fiber and protein contents are the achieved goals for lower caloric products to reach a better nutritional profile [18,53]. Moreover, the interference of mushrooms with the integrity of starch granules results in reduced starch susceptibility to digestive enzymes, providing a lower glycemic index [55]. According to the authors, the dietary fiber of mushroom powder disturbed the starch granule structures, making them irregularly shaped and reduced in their diameters, resulting in less susceptibility to enzymatic hydrolysis [55]. This means that the conversion rate of starch to glucose becomes slower and insoluble dietary fiber delays intestinal glucose absorption, benefiting the diets of diabetic individuals by lowering their postprandial blood glucose level. The decrease in starch hydrolysis index was also reported by Rathore et al. [59], with good nutritional, antioxidant properties and sensorial profiles, when *Calocybe indica*, known as milky white mushroom, was introduced in cookies replacing 10% of wheat flour.

*Lentinula edodes* has also been used to replace rice flour in gluten-free formulations, such as rice noodles [60] or muffins [61]. While the inclusion of rich-β-glucans in noodles increased the thermo-mechanical properties in the dough system producing noodles with increased extensibility and firmness, the inclusion of whole shiitake mushroom in muffins decreased the volume of the product with an increase in hardness [60,61]. Although the color and texture of the muffins were affected, sensorial parameters were still acceptable at 5% substitution. The rice flour replaced by *Volvariella volvacea* and *Pleurotus pulmonarius* in brown rice extruded snacks [62,63] and achieved good hedonic scores when a salted seasoning was added, although the texture and color of snacks were altered.

Several species (*Agaricus bisporus*, *Lentinula edodes*, and *Boletus edulis*) were introduced in pasta [64,65] and extruded products [66], replacing up 15% of semolina. In the case of pasta, the flour replacement with shiitake and *Agaricus bisporus* resulted in swelling and water indexes, as well as a moisture content similar to control samples made only with durum wheat semolina. However, shiitake addition resulted in pasta with the highest firmness and tensile strength. In their work of 2018, authors [65] reported good antioxidant properties in mushroom added samples (10% and 15%) and a decrease in the glycemic response. In extruded snacks, the mushroom addition resulted in less expanded products with lower water absorption index and altered microstructure characteristics. *Agaricus bisporus* were also used in sponge cake with good results at 10% of flour substitution [18] and 10–20% of flour substitution [67].

### 2.4. Salt Replacer

Sodium chloride (NaCl) is the most used ingredient to increase or enhance taste, flavor, and saltiness, improving palatability in food products, such as soups, bread, meats, sauces, and snacks [68]. Additionally, sodium chloride has a technological function in the elaboration of foods suchas meat products [69]; it solubilizes myofibrillar proteins for fat and water binding capacity in meat batter [70]. However, there is no doubt that, nowadays, sodium consumption exceeds the healthy recommended values established by the World Health Organization (5 g of salt/day or 2 g of sodium/day) [71]. Abundant literature has shown the health problems associated with a high dietary sodium intake, such as hypertension, cardiovascular diseases, stroke, and diet-associated diseases [72], and a limitation of salt intake is a necessary measure.

In the last decade, many countries around the world have launched strategies and programs to implement a gradual salt reduction in food products to positively modify the eating habits of consumers [73]. Most of the initiatives have encouraged a reformulation of foods through voluntary or mandatory salt level targets [74]. However, promoting a reduction of sodium content in food is a challenge because not only sensorial but also technological aspects can also be modified [68]. The partial replacement of NaCl with other salts, such as potassium chloride (KCl) or calcium chloride (CaCl_2_), has been a feasible alternative [69]. But the use of these salts is generally accompanied by certain bitter and metallic notes in the taste of the products [75].

Another alternative has been the use of monosodium glutamate (MSG) to enhance the perceived saltiness through the umami taste. Umami taste can regulate the appetite and improve satiety [76]. Umami, which means “delicious savory taste”, was coined in 1909 by Japanese chemist Kikunae Ikeda [77]. It is recognized as the fifth basic taste after sweet, sour, salty, and bitter taste. Umami has the property of enhancing food palatability modulating sweet taste, enhancing salty taste, and suppressing sour and bitter tastes [78]. Glutamic acid and its salt, MSG, are the main representative compounds and are widely used in the food industry, but they are not the only ones. Umami substances are naturally found in a variety of foods, including meat, cheese, seafood, vegetables, as well as in mushrooms [79]. Mushrooms are a natural source of umami compounds, such as free amino acids (glutamic and aspartic acids) and 5′-nucleotides such as 5′-guanosine monophosphate (5′-GMP), 5′-inosine monophosphate (5′-IMP), and 5′-xanthosine monophosphate [80,81]. According to Zhang et al. [82], L-glutamic acid and its sodium salt could be considered umami agents since they show umami taste while 5′-ribonucleotides do not produce umami taste directly, thus being considered umami enhancers. Abd El-Aleem et al. [83] extracted 5′-nucleotides (5′-IMP, 5′-GMP and 5′-XMP) from *Agaricus bisporus*, with 5′-IMP being the most abundant, but the extract showed a better enhancing effect in beef soup than the pure 5′-IMP, which was attributed to the synergistic effect between nucleotides. Li et al. [84] reported that 5′-AMP and 5′-GMP were the flavor nucleotides more abundant in *Lentinula edodes*. Besides glutamate and nucleotides, several peptides have also been demonstrated to have an umami taste. Other molecules with flavor-enhancing properties are generated through the Maillard reaction (a reaction between amino acid/peptides and reducing sugars), the so-called “Maillard reacted peptides” from the reaction of amino acid/peptides with reducing sugars [85].

The umami flavor of mushrooms offers a dual opportunity to improve the nutritional profile of foods by adding dietary fiber, protein, minerals, and bioactive compounds to foods but also imparting flavors that can complement and enhance the saltiness perception [86]. Reducing salt application in culinary preparations of meat by using the umami properties of *Agaricus bisporus* without compromising the flavor profile or consumer acceptance was initially investigated by Guinard et al. [26] and Myrdal-Miller et al. [25]. *Agaricus bisporus* (white button and portobello) mushrooms were also used in taco filling blends by Wong et al. [27], allowing reductions of 45% of salt content, although color modification with portobello could discourage its use (Table 1). This research group (Wong et al. [24]) also tested the replacement of meat by several concentrations of fresh and ground *A. bisporus*, reducing 25% of salt in beef patty formulations. The product where salt was partially replaced with mushroom received similar overall liking scores to its all-meat, full sodium counterpart. Recently, dos Santos et al. [87] reported that the glutamic acid from champignon mushroom was able to substitute the use of salt in a culinary preparation of chicken stroganoff during its preparation, allowing a higher than 50% reduction of NaCl and a good sensorial acceptance. Several works of Cerón-Guevara et al. [44,45,46] were successful in the replacement of 50% of salt by *Agaricus bisporus* and *Pleurotus ostreatus* in meat products.

The high potential of mushrooms to reduce sodium in meat products due to an excellent flavor-enhancing property may be tarnished by its impact on the technological properties of the food products. For example, the inclusion of 20% of fresh *Agaricus bisporus* as meat extender combined with eggplant powder, to replace up to 40% of salt in chicken nuggets was only suitable for salt reductions of 13%, in order to enhance the cooking yield, shrinkage, and firmness [75]. According to the authors, higher salt reductions decreased the concentration of extracted protein and weakened the protein–protein gel emulsion matrix, thereby reducing the ability to hold water and increasing cooking losses and shrinkage.

When an umami ingredient prepared from *Lentinula edodes* byproduct aqueous extract was introduced in low salt corn extruded snacks (with a salt reduction over 70%), the reduction of salt was sensorially noticed by consumers, but the low salt product was not sensorially rejected [88,89]. According to these authors, the umami ingredient proved to be efficient as a flavor enhancer, presenting similar results to MSG. However, Tepsongkroh et al. [63] reported that the substitution of 15% of the base formulation in brown rice snacks by *Pleurotus pulmonarius* needed to be improved through savory seasoning (8% of salt) addition to cover saltiness expectations.

The addition of 20% of water extracts from *Lentinula edodes* (mushroom homogenate) was the best option to reduce 50% of salt content in beef patties, although this concentration was not enough to enhance the salty perception in 75% salt reduced items [90].

The ability of mushrooms to replace salt seems to depend not only on the species, growth conditions and storage, but also on the technological processes to prepare the ingredient. Blanching, a common pretreatment applied to the mushrooms to avoid unwanted browning, has also been reported to modify the content of umami compounds with varied results [80]. According to Hu et al. [31] the hot air drying method presented a better potential to produce umami concentration in *Stropharia rugoso-annulata* mushroom compared to natural air drying or vacuum freeze-drying. On the other hand, variable results were found by Zhao et al. [91], who reported that vacuum drying of *Suillus granulatus* resulted in higher content of 5′-nucleotides and equivalent umami concentrations compared to natural-air, hot-air, and freeze-drying. Other preservation techniques can modify the flavor of the mushroom. For example, the fermentation of *Pleurotus eryngii* with selenium-enriched *Lactobacillus plantarum* produced the highest umami intensity, comparable to natural fermentation [92].

### 2.5. Other Additives Replacer

In meat products, phosphates are generally added to formulations to improve water-holding capacity by increasing the pH. Thangavelu et al. [93] indicated that the good water holding capacity and cook yield showed by *Agaricus bisporus* in some emulsioned meat products [94] made this species a good candidate as a potential replacer of phosphates in meat emulsions. When winter mushroom powder (*Flammulina velutipes*) was added in emulsion-type sausages to replace phosphates (Table 1), percentages of 1% increased the pH and inhibited the oxidation, while color and sensory properties remained unaltered [95]. Similar results were obtained by Jo et al. [96], who observed an increase in pH whereas the color and sensory properties were not negatively affected with 0.5% and 1% of *Flammulina velitupes* in low salt chicken sausage. However, the texture was modified, providing softer sausages while lipid oxidation was inhibited. In a later work by this group [97], plasma-treated *F. velutipes* at 1% showed to be a good nitrite replacer in ground ham, but not so effective in the total replacement of phosphates. Several concentrations (0–6%) of *Lentinula edodes* were sensorially investigated as total phosphate replacers in pork patties, with an increase in juiciness and decreased toughness and rubberiness of the pork [98].

Mushrooms have been proposed even as MSG replacers, considering the recent negative health perception attributed to this additive. Wang et al. [99] initially considered mushroom concentrate powder could be a potential replacer of MSG in chicken soup. However, compared with yeast extract, the mushroom concentrate failed to increase the salty taste in a later study, despite the perception of the “natural” label associated with this additive [100]. Cooked *Agaricus bisporus* was a good replacer for tomato pulp in the elaboration of ketchup with improved nutritional characteristics and good preferences in 50/50 mushroom/tomato formulations [101]. Likewise, this species was also employed to replace egg white, commonly used as a binder for plant-based meat substitutes, presenting positive hedonic scores, although steaming preparation of mushrooms was preferred over roasting [102]. Egg white is widely used as a binder in plant-based meat substitutes, contributing to the texture of the products during consumption [103]. For that purpose, Du et al. [102] elaborated mushroom-white egg patty prototypes with 10%, 20%, and 30% of white and crimini mushrooms to evaluate several sensory attributes. The addition of mushroom was acceptable up to 20%, with the steam method (water vapor at 163 °C for 10 min) and crimini mushroom being the most preferred.

**Table 1:** Recent works with edible mushroom species as food ingredient/additive replacers (last 5 years).

Ingredient/Additive Substitution	Mushroom Species	Food Product	Main Effects on Properties	Reference
Meat substitution (10%, 20%, 30%, 40%, 50%) Salt reduction (25%)	*Agaricus bisporus* F	Beef patties	Good overall liking, aroma, flavor, saltiness, and juiciness in 20% mushroom formulation	[24]
Meat substitution (15%, 30% and 45%) Salt reduction (15%, 30%, and 45%)	*Agaricus bisporus* (white button and portobello) C	Taco filling	Taco filling with 45% reduction of meat and salt was preferred over the full salt formulation	[27]
Meat substitution (25%, 50%, 75% and 100%)	*Lentinula edodes* D	Pork meat sausage	Increase in moisture, fiber, methionine, glutamic, cysteine and total phenolic content, cooking loss and antioxidant activity. Slight darkening of sausages and all formulations were sensorially acceptable but 25% replacement exhibited best characteristics	[37]
Meat substitution (2%, 4% and 6%)	*Flammulina velutipes* steam wastes D	Goat meat nuggets	Increase in water holding capacity Increase in oxidative stability No negative impact in color or sensorial properties 4% recommended substitution	[38]
Meat replacement (10%, 20% and 30%)	*Pleurotus sapidus* waste D	Chicken patties (65% meat)	10% optimal formulation Increase in hardness and chewiness	[39]
Fat substitution (25%, 50% and 75%)	*Agaricus bisporus* (5%, 10% and 15%), C	Beef burger (20% fat)	Good overall liking Less hard and chewy products and lower cooking loss Modification of color Good antioxidant properties at 15%	[17]
Fat Substitution (100%)	*Pleurotus eryngii* (raw, boiled, deep fried, fried)	Pork sausages (17% fat)	83–90% fat reduction Increase in protein, moisture, dietary fiber, cooking loss, water holding capacity Deep-fried and fried mushroom provided better odor, flavor, texture an overall acceptability	[43]
Fat reduction (30% and 50%) Salt, caseinate, phosphates reduction	*Agaricus bisporus* *Pleurotus ostreatus* (2.5% and 5%) D	Frankfurter (25% fat, 1.5% salt, 2% sodium caseinate, 0.5% phosphates)	Modification of color and texture: Agaricus gave darker and *Pleurotus* softer samples No antioxidant effect observed Mushroom samples were sensorially acceptable with better scores at low mushroom level	[44]
Fat reduction (25% and 50%) Salt reduction (50%)	*Agaricus bisporus* *Pleurotus ostreatus* (2.5% and 5%) D	Beef burger (10% fat and 1.2% salt)	Increase in protein and dietary fiber Lower increase in hardness compared to fat reduced control samples Slight antimicrobial effect in pseudomonads Sensorially acceptable, especially 2.5% mushroom samples	[45]
Fat reduction (30% and 50%) Salt, phosphates and nitrite reduction (50%)	*Agaricus bisporus* *Pleurotus ostreatus* (7.5% and 10%) D	Liver pate (30% fat 2% salt)	Increase in protein, dietary fiber and pH Color modifications Increase in hardness, adhesiveness	[46]
Fat reduction (25% and 50%) Salt reduction (37.5% and 75%)	*Agaricus bisporus* (15% and 30%) C	Beef burger (20% fat and 2% salt)	Increase in moisture, no color modification, lower hardness 15% mushroom presented the lowest lipid oxidation Sensorial profile affected by mushroom and salt content	[49]
Cake flour reduction (5%, 10% and 15%)	*Agaricus bisporus* D	Sponge cake	Increase in protein, minerals, increase in viscosity, darker color, 10% better sensory characteristics	[18]
Wheat flour (10%, 20% and 30%)	*Pleurotus sapidus* waste (10%, 20% and 30%) D	Steamed buns/Cookies	10% optimal formulation Increase in hardness and chewiness	[54]
Wheat flour substitution (4%, 8% and 12%)	*Pleurotus sajor caju* D	Biscuits	Glycemic response reduced by interference of mushroom in integrity of starch granules. Addition of 8% resulted in non-significant sensorial differences with control.	[55]
Wheat flour substitution (2%, 4%, 6%, 8% and 10%)	*Pleurotus ostreatus* D	Noodles	Increase in protein and fiber content More than 4% increased cooking time, water absorption and tensile strength Good acceptability with noodles at 4% replacement.	[56]
Wheat flour substitution (5%, 8% and 10%)	*Pleurotus ostreatus* D	Noodles	5% resulted in better sensory scores than higher replacements. Improved nutritional characteristics	[57]
Wheat flour substitution (5%, 10%, 15% and 20%)	*Calocybe indica* D	Cookies	Increase in protein, dietary fiber, β-glucans and antioxidant properties 10% replacement gave acceptable sensory scores despite the diminution in textural properties	[59]
Rice flour substitution (5%, 10% and 15%)	*Lentinula edodes* D	Muffins	Decrease in volume, higher weight ant hardness Color modification with mushroom. Improvement of antioxidant properties. Comparable and better consumer acceptability than control	[61]
Brown rice flour substitution (10%, 15% and 20%)	*Volvariella volvacea* D	Brown rice extruded snacks	Higher nutritional values and increase in phenolic content and antioxidant activity Decrease in expansion rate, lightness, and texture hardness Optimum: 14–17% of mushroom powder	[62]
Formula base replacement (15%) Sal reduction (no seasoning added)	*Volvariella volvacea* and *Pleurotus pulmonarius* D	Brown rice extruded snacks with/without seasoning	Mushroom and seasoning added snacks presented high hedonic scores and positive emotions *Pleurotus pulmonarius* and seasoning better evaluated in saltiness scores than control and samples with no seasoning	[63]
Durum wheat semolina substitution (5%, 10% and 15%)	*Agaricus bisporus*, *Lentinula edodes*, *Boletus edulis* D	Pasta	Increase in pasta firmness and increase in cooking loss Reduced glycemic response with increase mushroom concentration Increase in phenolic content and antioxidant capacity	[64,65]
Durum wheat semolina (5%, 10% and 15%)	*Agaricus bisporus*, *Lentinula edodes*, *Boletus edulis* D	Extruded products	Decrease in water absorption, moisture, total starch content, reduced degree of starch gelatinization Higher antioxidant values and phenolic compounds	[66]
Wheat flour substitution (10%, 20%, 30%, 40% and 50%)	*Agaricus bisporus* D	Sponge cake	Not significant differences in organoleptic properties with control up to 20% of addition	[67]
Salt reduction (0–40%)	*Agaricus bisporus* F	Chicken nuggets (1% salt)	Low salt reduction (13%) combined with eggplant enhanced cooking yield, shrinkage, and firmness. Diminution of physical parameters when salt content was reduced.	[75]
Salt reduction (~7%)	Umami ingredient *Lentinula edodes* (1–1.5%) WE	Corn extruded snacks (2.8% salt + 0.6% monosodium glutamate MSG)	Umami ingredient similar performance to monosodium glutamate (MSG) flavor enhancer	[88,89]
Salt reduction (50% and 75%)	*Lentinula edodes* water extract (5%, 12.5% and 20%) WE	Beef patties (1.3% salt)	Extract (20%) was effective as taste enhancer in salt reduction of 50% with better acceptance in color, aroma, texture, flavor, and overall impression.	[90]
Phosphate replacer	*Flammulina velutipes* (0.5%, 1%, 1.5% and 2%) D		Inhibition of lipid oxidation, no color or sensory modification up to 1.5% concentrations	[95]
Phosphate replacer	*Flammulina velutipes* 0.5% and 1% D	Low salt chicken sausage (0.3% sodium pyrophosphate)	Inhibition of lipid oxidation Softer texture No color or sensory alterations	[96]
Nitrite/Phosphate (total replacement)	*Flammulina velutipes* (winter mushroom) 1% D	Ground ham (48 mg nitrite/kg and 3 g/kg phosphates)	Good source of nitrite No complete replacement of phosphates in cured meat products	[97]
Phosphate replacer	*Lentinula edodes* (0–6%) D	Pork patties (0.5% phosphate)	Shiitake mushroom increased juiciness and decreased toughness and rubberiness.	[98]
MSG substitute	Mushroom concentrate (0.1%)	Reduced salt chicken soup (0.4% salt/0.1% MSG)	Mushroom concentrate failed to increase the salty taste. Samples were less preferred compared to MSG or yeast extract	[99,100]
Tomato pulp reduction (25%, 50%, 75% and 100%)	*Agaricus bisporus* (25, 50, 75 and 100%) C	Mixed ketchup	Mixture 50% mushroom/50% tomato pulp the best preferred. Increase in protein, fiber, and ash content	[101]
Egg white replacer (10%, 20% and 30%)	*Agaricus bisporus* (white and crimini) (steamed and roasted)	Mushroom/egg white patty	Positive hedonic scores except in overall acceptance of roasted mushroom patties Steamed crimini 20% patty most preferred.	[102]

In mushroom species column: D: dried; F: Fresh, C: cooked, WE: aqueous extract.

## 3. Use of Edible Mushroom with Antioxidant and Antimicrobial Purposes

Several functional properties attributed to medicinal mushrooms are related to the presence of antioxidant substances, such as phenolic compounds and indole compounds, and carotenoids [104]. Phenolic acids, such as chlorogenic, gallic, caffeic, protocatechuic, and syringic acids are commonly found in mushrooms and several works have reported their content in different mushroom species [105,106]. Indole components include melatonin or indole derivatives, such as L-tryptophan [107]. Some water-soluble vitamins (vitamins B and C), as well as lipid-soluble vitamins (tocopherols and vitamin D) have been found in edible mushrooms contributing to the antioxidant properties. Other organic compounds with antioxidant activity are ergothioneine, benzoquinones, and some minerals, such as selenium, zinc, manganese, magnesium and copper, are also involved in the antioxidant activity [104,108].

Considering the reported antioxidant properties for edible mushrooms [109,110], the addition of mushrooms (fresh, dried or extracts) has been successful in preventing or reducing lipid oxidation, especially in products with high levels of fat, as alternatives to synthetic antioxidants. In several works where mushroom species were used to replace main ingredients (Table 1), the antioxidant effect in the food product was also investigated, generally by determining thiobarbituric acid reactive substances (TBARS) or peroxide value, which measure oxidation products such as malondialdehyde and peroxides, respectively [17,38,49,62,66,96]. For example, in the case of meat products, Pil-Nam et al. [111] reported that 1.2% of shiitake mushroom freeze-dried powder included in frankfurters kept TBARS at 0.48–0.50 mg MDA/kg sample during cold storage, much lower than the values (almost 3.0 mg MDA/kg after 30 days storage) of control samples added with 100 mg/kg NaNO_2_ (Table 2). The mushroom powder was significantly more effective at 1.2% in preventing lipid oxidation than NaNO_2_, which is reported to have antioxidant activity in comminuted meat products. Moreover, mushroom concentrations of 0.8 and 1.2% inhibited the growth of aerobic counts. DPPH (2,2-diphenyl-1-picrylhydrazyl), ABTS (2,2′-azino bis (3-ethylbenzo-thiazoline-6-sulphonic acid)), FRAP (ferric reducing antioxidant power) assays and total phenolic compounds are also techniques commonly applied in mushrooms and extracts to evaluate the antioxidant effect [110].

Related to *Agaricus bisporus*, this mushroom species was added at low concentrations to a Turkish dry-fermented product by Gençcelep [112] and to ground beef by Alnoumani et al. [113] with antioxidant purposes (Table 2). Both works reported lower TBARS in mushroom added samples. Even in ground beef, the antioxidant capacity of dried *Agaricus bisporus* increased with the highest salt concentration (1.5%), presenting better TBARS values than the samples with 0.1% of the natural antioxidant rosemary [113]. The addition of low percentages of dried *Pleurotus ostreatus* (1%, 2% and 3%) resulted as well in lower TBARS values in beef salami but with good sensory results comparing to control samples [114]. Wang et al. [115] reported a 10-fold diminution of peroxide values in Cantonese sausage with 4% *Volvariella volvacea* although the product was less sensorially appreciated than sausages with 2% of mushroom. The same research group also observed a remarkable antioxidant effect of fresh *Flammulina velutipes* at 2.5% with better odor and taste than the use of dried mushrooms [116]. Higher concentrations of *Auricularia auricula* (10–15%) have led to higher antioxidant capacity values (DPPH) correlated with total phenols in brown rice extrudates compared to control samples [117]. *Cordyceps militaris* has exhibited better TBARS and DPPH (results in a Korean chicken soup at low concentrations (2–3%) compared to control samples [118].

When the mushroom is added fresh or dried in food to control lipid oxidation, a modification of technological and sensorial properties is unavoidable, especially color. This effect can be overcome by adding extracts. For example, Barros et al. [119] reported good antioxidant properties of 1%, 3%, and 5% of *Boletus edulis* of lyophilized aqueous acetone extracts added to beef patties attributed to tocopherols, ascorbic and phenolic acids. Decoction extracts (0.75% and 1.0%) of this species were also used in frankfurters with modest antioxidant effects and total mesophilic bacteria inhibition [120]. The inhibition of lipid peroxidation was significantly lower compared to ascorbic acid or ɑ-tocopherol. These authors have previously used decoction extracts of *Cantharella cibarius* with similar results [121]. They considered that decoction was not enough to extract polyphenols responsible for the antioxidant effect.

Residues from a hydroalcoholic extract of *Agaricus blazei* exerted antioxidant activity in enriched Omega-3 milk, decreasing lipid oxidation and preserving the polyphenol bioavailability, which could offer antioxidant activity against the free radicals present in the human body [122]. Hydrophilic extract from *Flammulina velutipes* was also used to stabilize the fresh color of bigeye tuna at 1%, 3%, and 5%, thereby extending the ice storage and being a more effective extract at 5% than other antioxidants such as ascorbic acid, and alfa tocopherol at 500 ppm [123].

**Table 2:** Antioxidant, antimicrobial and sensorial effects of the incorporation of edible mushrooms in food products.

Mushroom Species	Food Product	Antioxidant/Antimicrobial Effect	Reference
*Lentinula edodes* (0.8–1.2%) D	Frankfurter	Antioxidant stability during storage Lower TBARS than the control with NaNO_2_ Inhibition of aerobic bacteria counts Better flavor, taste, texture, and acceptability scores	[111]
Dried *Agaricus bisporus* (0.5%, 1% and 2%) D	Sucuk (Turkish dry fermented product)	Mushroom x ripening period affected to lipid oxidation parameters Color parameters affected by mushroom powder	[112]
*Agaricus bisporus* powder 1%, 2% and 4% D	Ground beef (0, 1 and 1.5% salt)	Inhibition of lipid oxidation independently of sodium chloride concentration added Better TBARS values than 0.1% rosemary	[113]
*Pleurotus ostreatus* (1%, 2% and 3%) D	Beef salami	Good antioxidant properties No negative effect on sensory properties	[114]
*Volvariella volvacea* (1%, 2%, 3% and 4%) D	Cantonese sausage	10-fold reduction of lipid peroxide (4%) Increase in amino acids and volatile compounds Better acceptability	[115]
*Flammulina velutipes* (fresh & dried) (2.5% and 5%) F, D	Cantonese sausage	Reduction of peroxide value and TBARS More effective with fresh mushroom	[116]
*Auricularia auricula* (5%, 10% and 15%) D	Brown rice extrudates	10–15% higher phenolic and antioxidant effect Lower glycemic load after ingestion	[117]
*Cordyceps militaris* (0–3% F, D)	Samgyetang (Korean chicken soup)	Antioxidant properties at 2–3% of dried mushroom Enriched flavor (increase in 5′-AMP, L-glutamic and L-aspartic acids)	[118]
*Boletus edulis* (0.75% and 1.5%) Decoction Extract	Frankfurter (25% fat)	Microbial inhibition of total aerobic mesophilic bacteria Lipid stability Color modification, hardness increase	[120]
*Cantharellus cibarius* (0.75% and 1.5%) Decoction Extract	Frankfurter (25% fat)	Antimicrobial activity against total aerobic mesophilic bacteria Weak radical scavenging activity (ABTS) Improved technological and sensory properties	[121]
*Agaricus blazei* (0.1%, 0.2% and 0.3% Hydroalcholic Extract)	Enriched Omega-3 milk	Good antioxidant activity and polyphenols are bioavailable	[122]
*Lentinula edodes* by-products (0.3% and 0.6%) Water extract/Ethanolic extract	Fermented sausage	Ethanolic extracts improved lipid oxidation and showed antimicrobial activity against pathogens (*Sthaphylococcus aureus*, *Listeria monocytogenes*, *Eschirichia coli*, *Salmonella typhimurium*)	[124,125]

In mushroom species column: D: dried; F: fresh; E: extract.

Water extracts from *Lentinula edodes* added at 0.6% showed better effects than a synthetic antioxidant (BHT) in a fermented sausage, improving lipid oxidation and microbial stabilities, and inhibiting the growth of pathogens such as *Staphylococcus aureus*, *Listeria monocytogenes* and *Escherichia coli O 157* (Table 2). In addition, no defects in color, texture and sensory quality were appreciated in the product [124]. This research group reported better antioxidant and antimicrobial results with the ethanolic extract compared to BHT or nitrite/nitrate, and also inhibited *Salmonella typhimurium* [125]. The antimicrobial activity of edible mushrooms in foods has been less thoroughly studied and the interest of mushrooms as an antimicrobial agent has been more focused on the formulation of nutraceuticals by means of spray-drying or freeze-drying to have a potential effect in the regulation of the gut microbiota rather than their utilization as preservatives in foods [126].

Concerning ergothioneine (a thiourea derivative from histidine), only fungi and certain bacteria can produce it [109]. The antioxidant effect of ergothioneine from *Flammulina velutipes* has been proved to be efficient in preventing oxidation reactions in fish and seafood. Bao et al. [127] reported that 3.03 mg/mL of ergothioneine from a hydrophilic extract inhibited the formation of metmyoglobin in bigeye tuna fish. In a later work of these authors, the extracts from *Flammulina velutipes*, *Lentinula edodes*, *Pleurotus cornucopiae* and *Pleurotus eryngii* showed notable results in preventing discoloration in minced bigeye tuna and yellowtail, with *Flammulina* extract presenting comparable efficiency to sodium ascorbate [128]. Different water extracts prepared from *Flammmulina velutipes* at 0.5% and 1%, rich in ergothioneine (2.05–9.1 mg/mL), were applied by immersion to cultured Pacific white and black tiger shrimps and crab to prevent post-mortem melanosis by decreasing the expression of prophenoloxidase gene and phenoloxidase activity [129,130,131].

It is worth noting that processing techniques employed to condition the raw material can affect the content of bioactive substances. According to the review of Yadav and Negi [15], blanching and boiling generally reduce 31–79% of the total phenols affecting the antioxidant activity. Roncero Ramos et al. [132] also reported a decrease in antioxidant activity after boiling and frying processes. However, the study of Hwang et al. [133] indicated that roasting temperatures of 140 °C applied to *Lentinula edodes* increased antioxidant activities, including ABTS and DPPH radical scavenging activity, and total phenols and polyphenols contents. The authors attributed the increase in antioxidant activity to the deactivation of endogenous enzymes responsible for oxidation. The oven-drying of *Agaricus bisporus* at 60 °C for 20 h increased 64.15% of total phenolic compounds, and the processed mushroom was more effective than the lyophilized powder in reducing the TBARS when they were applied to ground beef [134]. Likewise, antioxidant, nutraceutical and bioactive properties of mushroom could be enhanced by enzymatic hydrolysis as was described by Goswani et al. [135], by hydrolyzing *Pleurotus ostreatus* protein with proteinase K. The fermentation of *Pleurotus eryngii* with selenium-enriched *Lactobacillus plantarum* also improved the antioxidant properties by a positive effect on total phenolic content and DPPH radical scavenging activity [92]. Hot water and supercritical CO_2_ have been successfully used to obtain extracts rich in antioxidant polysaccharides, as described by Barbosa et al. [136]. These findings open the door to new uses of components extracted from mushrooms as functional ingredients in food products.

## 4. Conclusions

The published studies confirm the promissory potentialities of edible mushrooms, especially to replace main ingredients in several foods, such as meat, fat, flour and salt. Although the ability to reduce phosphates, nitrites or synthetic antioxidants have been less studied, results are encouraging. Considering the high number of edible mushroom species, the possibilities are endless, provided the processes of using mushrooms and their byproducts are feasible. However, when mushrooms are incorporated into food, their functionality should be previously analyzed since several properties related to technological aspects, such as emulsifying, gelling, water and fat binding abilities, are generally worse than those from the replaced ingredient limiting their use. Furthermore, sensorial properties are usually modified and might not be welcome for a non-familiarized consumer with this specific taste. In this regard, the incorporation of moderate concentrations should be the pattern to introduce mushrooms in foods, even though more studies about extraction and modification of specific components should surpass these limitations.

## Data Availability

All data are presented in the manuscript.
